# Pain in older adults with dementia: improving diagnosis to provide better care

**DOI:** 10.1055/s-0043-1772671

**Published:** 2023-08-30

**Authors:** Paulo Caramelli

**Affiliations:** 1 Universidade Federal de Minas Gerais, Faculdade de Medicina, Unidade de Neurologia Cognitiva e do Comportamento, Belo Horizonte MG, Brazil

Pain is a highly frequent health problem in older adults. In a large and recently published study conducted in Brazil with 9,234 community-dwelling individuals aged 50+ years, the prevalence rate of recurrent pain was 36.9%. 1 As might be expected, pain is also very common among people with dementia. A nationwide cross-sectional investigation from France reported a prevalence of 57.7% of chronic pain in individuals with Alzheimer's disease and related dementias, a frequency that was higher than the prevalence of chronic pain among subjects without dementia (49.9%). 2

Acute and chronic pain can cause prominent behavioral changes (e.g., agitation, aggressiveness, mood disturbances) and further impair cognitive functioning in individuals with diagnosis of dementia, negatively interfering with their quality of life and of their caregivers. Moreover, a significant proportion of subjects with pain use opioid drugs (e.g., 30% within the last three months in the Brazilian study 1 ), a drug class that may negatively impact cognitive performance. 3 This latter feature is of special concern in the dementia population.

Within this common clinical scenario, it is of utmost importance to effectively detect and quantify pain in people with dementia. However, the cognitive decline manifested by these individuals, particularly the language and memory deficits, poses significant difficulties for an adequate assessment with the self-report tools that are generally used for this purpose. 4 Hence, the development and validation of instruments suitable for detecting and measuring pain in dementia is of great clinical relevance.

The Pain Intensity Measure for Persons with Dementia (PIMD) has been developed to specifically assess and measure pain in this clinical context. 5 The instrument was derived from a large list of pain indicators of previously existing tools and the items which were considered the best predictors of physical pain, using the Delphi Method, were selected. Seven indicators assessing the presence and intensity of pain were included in the PMID, namely, facial expressions (three indicators), body positioning, muscle stiffness, sighing, and verbal complaints (one indicator for each variable). A study conducted with individuals with moderate and advanced dementia living in long-term care facilities in the USA confirmed the adequate validity and reliability of the PIMD. 6

In this issue of *Arquivos de Neuro-Psiquiatria* , Foraciepe et al. 7 present the validation of the Brazilian version of the Pain Intensity Measure for Persons with Dementia (PIMD-p), providing a valuable contribution to the assessment of pain in people with dementia in Brazil. The investigators evaluated 50 older adults (mean age of 86.1 years) with diagnosis of dementia (88% of whom with moderate or severe dementia), recruited either at an outpatient clinic or at long-term care facilities. Almost half of the sample presented moderate or severe pain, intensity ranges that are the most burdensome and consequently demand an earlier diagnosis. The PIMD-p displayed good internal consistency, and high intra- and inter-rater reliabilities. As for convergent validity, most pain indicators strongly correlated with pain intensities evaluated by caregivers and nurses. Finally, the authors showed that PIMD-p was highly accurate for pain measurement (area under the ROC curve = 0.93), also defining a cut-off score for the detection of moderate/intense pain, which showed elevated specificity (95.7%).

The PIMD-p thus proved to be a valid instrument for the detection and quantification of pain in the context of dementia in Brazil. The use of the PIMD-p in clinical settings may prompt a better recognition of this common symptom and assist healthcare professionals to provide better care, to avoid iatrogenesis, and help to improve the quality of life of persons with dementia.
